# The Neural Markers of Perceptual Uncertainty/Curiosity—A Functional Near-Infrared Spectroscopy Pilot Study

**DOI:** 10.3390/brainsci15040411

**Published:** 2025-04-18

**Authors:** Adrian Korniluk, Barbara Gawda, Małgorzata Chojak, Anna Gawron

**Affiliations:** 1 Department of Psychology of Emotion & Personality, Maria Curie-Sklodowska University, 20-612 Lublin, Poland; adrian.korniluk@mail.umcs.pl; 2 Neuroeducation Research Lab, Maria Curie-Sklodowska University, 20-612 Lublin, Poland; malgorzata.chojak@mail.umcs.pl (M.C.); anna.gawron@mail.umcs.pl (A.G.)

**Keywords:** perceptual uncertainty, fNIRS, curiosity, orbitofrontal cortex

## Abstract

**Background:** Curiosity is an immanent aspect of human experience linked to motivation, information-seeking, and learning processes. Previous research has highlighted the significant role of the orbitofrontal cortex (OFC) in curiosity-driven behaviors, particularly in processing uncertainty and evaluating information. **Methods:** This study aimed to examine cortical activation during the induction of perceptual uncertainty using a modified blurred picture paradigm. A total of 15 participants were tested with fNIRS (functional near-infrared spectroscopy) while viewing pairs of images designed to induce perceptual uncertainty. **Results:** The results revealed a differential hemodynamic response in one of the analyzed channels associated with orbitofrontal cortex activation, with higher activity when uncertainty was reduced (the matching condition compared to the non-matching condition). **Conclusions:** These findings confirm the existence of neural pathways of curiosity. Furthermore, our study also highlights the spatial limitations of fNIRS in precisely localizing OFC activity.

## 1. Introduction

Curiosity is a multifaceted psychological phenomenon that constitutes a fundamental aspect of human cognition [1]. It is defined as a psychological state that includes recognition of an information gap, combined with a sense that closing the gap is possible and intrinsically valuable [2] (p. 909). In a classical framework, the state of arousal known as curiosity can be elicited by ambiguous, complex, or contradictory stimuli and satisfied through access to relevant and desired information [3]. This state is called perceptual curiosity, which is distinguished from epistemic curiosity, which closely resembles a personality trait. Curiosity as a personality trait is related to openness to experiences [3,4].

It is possible to broadly identify a number of functions that curiosity can play. Key ones include exploratory, motivational, protective, and developmental roles. For this reason, curiosity remains a significant topic in contemporary research. Regarding its exploratory role, curiosity guides exploration in the context of learning and knowledge acquisition [5]. Curiosity activates a willingness to seek out knowledge and new experiences and a willingness to embrace the novel, uncertain, and unpredictable nature of life [6].

Curiosity has a motivational nature [6,7]. It is a motivational determinant of human behaviors associated with learning, decision-making, and other intentions [8]. Curiosity impacts motivation in two ways: firstly, by evoking a strong desire to understand, and secondly, by fostering endurance to delayed gratification [9]. It has been shown that it enhances learning efficiency [10,11]. Research demonstrated that prompting students to generate predictions, which involve uncertainty/curiosity regarding outcomes, positively impacts their learning [5]. In studies examining purchasing intentions, perceptual curiosity was found to moderate the relationship between pleasure and arousal responses (induced by the emotional climate of e-commerce websites such as color and graphics) and participants’ purchasing intentions [12]. Some researchers emphasize the metacognitive nature of curiosity, highlighting its two-step triggering process. In the work of Goupil and associates, it was proposed that curiosity is initiated by an evaluation of one’s own informational needs [13]. The anticipation of the probability that environmental exploration will yield significant informational benefits complements this process. Furthermore, despite the fact that individuals may be motivated by non-immediate gratification, they underestimate the motivational properties of curiosity [14]. It must be stressed that curiosity is a strongly rewarding emotion—when individuals are driven by curiosity, they undertake various activities not because they lead to some reward, but because they are themselves a reward. For this reason, according to many researchers, curiosity is the strongest driver of learning that nature has equipped people with [4,15].

Curiosity can play a protective role. In a broad context, it is regarded as a psychological resource or attribute that can be targeted through interventions [16], yielding benefits such as increased life satisfaction [17], reduced risk of depression symptoms [18], and protection against stress [19]. Curious people can develop better strategies to cope with stress due to their cognitive flexibility [20]. In this context, particularly from the perspective of positive psychology, curiosity is related to well-being [6,21] and is conceptualized as one of the strengths of character [22], while in philosophical discourse, it is described as an epistemic virtue [23]. Considering the protective role of curiosity, the importance of therapeutic interventions aimed at strengthening curiosity is emphasized [15,16].

Curiosity plays a crucial role in healthy human development. Research indicates that curiosity is associated with a willingness for personal development and a need for a sense of meaning in life [24]. Curiosity has a positive correlation with a sense of meaning in life [25]. Individuals with a curious disposition, through continuous exploration, can construct a positive view of the world that is explainable and well-structured. They set clear goals, which they then achieve, and this subsequently contributes to the development of meaning in life. Curiosity supports individuals in constructing a healthy self-image. Curiosity also helps to develop creativity, intelligence, and personality as it stimulates people, mobilizes their energy, and encourages them to act [15,19]. By focusing on novelty and challenge, people who feel curious stimuli construct their views of self, others, and the world with an inevitable stretching of information, knowledge, and skills. This movement toward intrinsically valued directions appears to be a pathway to the building of meaning in life, with the simultaneous presence of a positive present (mindful engagement and sense of meaningfulness) and future time orientation (searching for meaning and planning long-term goals with minimal worry about obstacles) and challenges [20,24,26]. Curious people develop themselves in a more flexible way [26].

### 1.1. Processing of Blurred Pictures

Perceptual uncertainty is a condition studied within the framework of perceptual curiosity [27]. It is induced by ambiguous stimuli and reduced through exploratory actions [28]. Stimulus uncertainty automatically induces attention [29,30].

At the operational level, this concept can be associated with object complexity. Studies indicate that the complexity of the stimulus/object can evoke “visual curiosity”, which enhances learning and memory [31]. The findings of Sun and Firestone [32] further demonstrated that an object’s perceptual complexity can encourage individuals to devote more attention to it and engage more deeply in its exploration. Simultaneously, complexity influences perception. In identification and categorization tasks with minimal differences between stimuli, reaction times are shorter compared to situations where differences are relatively large [33].

One of the methods of examining perceptual uncertainty involves the use of blurred photos/images. As early as the 1960s, behavioral studies focused on object recognition in blurred pictures [34]. Researchers identified blurred pictures as a potential tool for inducing perceptual uncertainty [35]. It was observed that low-pass spatial filtering in image processing affects subjective emotional assessments, whereas image size manipulation does not have a significant effect [36]. This same manipulation was later applied in a study utilizing startle EMG to assess physiological responses. It was found that both blurring and reducing the image size diminished affective modulation, as measured by skin conductance; however, the startle reflex—an automatic reaction to a strong stimulus—remained unchanged despite image degradation [37].

In-depth research on perceptual uncertainty yields applicable insights across multiple domains of scientific inquiry, including but not limited to decision-making [38] and learning [39]. Both areas have been extensively explored within the neuroscientific literature. For instance, in the domain of learning, the use of perceptual uncertainty as an experimental paradigm has contributed to a better understanding of the cortico–striatal–thalamic loop involved in the classification of distorted visual patterns [40]. Additionally, a theoretical neural circuit model has been proposed to account for decision uncertainty and change-of-mind processes [41], further highlighting the importance of this research perspective. In-depth research on brain models of perceptual uncertainty/curiosity is part of a neurocognitive trend searching for explanations of the neural mechanisms of cognitive processes associated with curiosity, such as attention processes, memory, learning, or executive functions. It will allow for a holistic insight into these complex mental phenomena by providing relevant data regarding the neural networks of curiosity and, consequently, the monitoring of this process; the possibility of arousing, developing, or reinforcing it.

### 1.2. Curiosity in Neuroimaging Studies

To date, several studies have attempted to describe the neural mechanisms of curiosity. In 2009, Kang and colleagues conducted an fMRI study that revealed that brain activity in the caudate nucleus and the inferior frontal gyrus (IFG) is associated with curiosity. These structures are activated in response to the anticipation of various types of rewards, suggesting that curiosity induces an expectation of reward [42]. Cervera, Zhe Wang, and Hayden [43] identified a network of neural structures potentially involved in curiosity, which includes the anterior cingulate cortex (ACC), inferior parietal lobule (IPL), orbital frontal cortex (OFC), lateral habenula (LHb), dopaminergic midbrain (DAM), hippocampus, and striatum. Activation of the nucleus accumbens (NAcc) in the context of curiosity was also observed during states of high curiosity in learning, indicating its connection to memory [44].

The relationship between curiosity and memory is also illustrated by the theoretical model. Gruber and Ranganath [45] proposed a framework encompassing a cycle of prediction errors, evaluation, curiosity, and exploration (PACE). In this model, curiosity arises when prediction errors occur (contextual—engaging the hippocampus; informational—engaging the anterior cingulate cortex). Subsequently, the lateral prefrontal cortex evaluates these errors in terms of valence, determining whether response inhibition or exploration will follow. This process shapes an experience that activates mechanisms associated with either anxiety (involving the amygdala) or curiosity (involving the dopaminergic system). The PACE cycle concludes upon solving the problem/reduction of uncertainty. Furthermore, Ligneul, Mermillod, and Morisseau [46] demonstrated that the rostrolateral prefrontal cortex (rlPFC) is associated with epistemic curiosity, while surprise—a state related to curiosity—is processed in the ventromedial prefrontal cortex (vmPFC).

Cohanpour, Aly, and Gottlieb [47] conducted a study assessing the certainty in identifying distorted images of animals and objects, as well as curiosity about seeing a clear version of the image. The findings revealed that the occipitotemporal cortex (OTC) was associated with processing uncertainty and an increase in curiosity, whereas activity in the ventromedial prefrontal cortex (vmPFC) and anterior cingulate cortex (ACC) was linked to gaining certainty and a decrease in curiosity.

In the study by Jepma et al. [27], with the use of the blurred pictures paradigm, increased activity was observed in structures sensitive to aversive conditions, including the anterior cingulate cortex (ACC) and anterior insula. In contrast, during the reduction/relief of perceptual uncertainty, greater activation was detected in cortical structures, specifically the orbitofrontal cortex (OFC) and lateral occipital cortex (LOC). This finding was later confirmed in subsequent research, which additionally demonstrated simultaneous activation of the ACC and the nucleus accumbens (NAcc) in response to both curiosity and fear [48]. NAcc activation in curiosity-related contexts has also been observed during states of high curiosity in learning, reinforcing its strong association with memory processes [44]. In the study by Van Lieshout et al. [49], parietal cortex activity was also observed during curiosity induction and uncertainty about outcomes, while a decrease in curiosity leads to the activation of the insula, orbitofrontal cortex, and parietal cortex.

### 1.3. Hypotheses

Considering these findings, it is reasonable to expect cortical activation to be observable through fNIRS during the reduction of perceptual uncertainty, specifically in the orbitofrontal cortex (OFC—associated with reward evaluation) and the inferior parietal lobule (involved in control functions related to curiosity) [27,43,49]. Notably, previous studies have investigated the activation of OFC using fNIRS, e.g., [50,51]; however, due to the spatial limitations of the method, the combined measurement of neighboring structures is indispensable.

This study assumes that in the process of reducing perceptual uncertainty, OFC structures may be involved in evaluating incoming information, while the inferior parietal lobule may be engaged in cognitive control and uncertainty processing. Comparisons will be made by contrasting (a contrast analysis allowing differences to be indicated) the options of increasing uncertainty and decreasing uncertainty.

The aim of our pilot study was to test an experimental procedure originally developed for fMRI and to adapt it to the fNIRS environment. This approach is grounded in a specific research framework and in hypotheses concerning the activation of particular brain structures. The successful triangulation of theoretical assumptions, existing knowledge about the function of these regions, and an informed awareness of methodological differences between neuroimaging techniques, considering the nature of this study, supports the interpretation that any observed effects, while potentially reflecting both a successful methodological transfer and the presence of genuine neural responses, are more plausibly attributable to the former, and only to a lesser extent to the latter.

## 2. Materials and Methods

This study presents the results of a pilot study based on the blurred pictures paradigm. The study is inspired by the work of Jepma and colleagues [27], with key modifications. Instead of fMRI, we used fNIRS for examining brain activation. Additionally, aspects of the procedure were altered, including epoch duration, inter-trial variability, and the number of presented stimuli.

**Figure 1:**
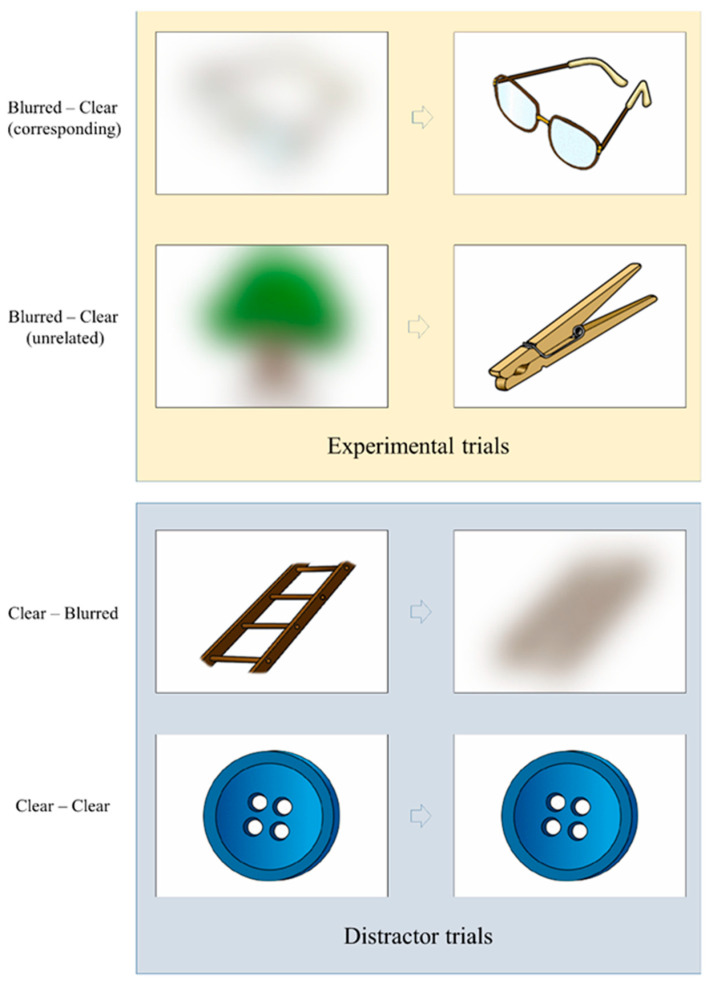
Schematic representation of the stimulus presentation procedure in the experiment.

In the original study, perceptual uncertainty was manipulated by presenting sequences of two images across four conditions (see Figure 1):

1. A blurred image followed by a matching clear image;
2. A blurred image followed by a non-matching clear image;
3. A clear image followed by a matching blurred image;
4. A clear image followed by a matching clear image.

For analysis, the first two conditions were compared after the presentation of the second image (to assess perceptual uncertainty reduction). The remaining two conditions served as distractors.

The images used in this study were sourced from the Rossion and Pourtois [52] dataset, which consists of colored images standardized for naming agreement and complexity. The images that were pseudorandomly drawn for this study are listed in Appendix A, Table A2. They were displayed on a computer screen using PsychoPy software (v2023.2.3), with the default light gray background. The data were collected using fNIRS technology, specifically the Scout model by NIRX (Berlin, Germany). The data were collected using two wavelengths (760 and 850 nm) to measure changes in oxygenated and deoxygenated hemoglobin levels.

### 2.1. Participants

A total of 15 participants aged from 21 to 30 years took part in the experiment, including 14 females and 1 male. All participants had at least a high school or higher level of education. None of them reported visual or neurocognitive impairments that could significantly affect their performance in the experiment (the screening interview).

The exclusion criteria for participation in this study (self-reported) are as follows:

- Medically documented history of brain damage (e.g., cerebral edema) and/or cranial implant(s);
- Medically documented history of neurological disorders (e.g., epilepsy) and/or mental illnesses (e.g., depression);
- Uncorrected visual impairment;
- Fine motor difficulties (e.g., difficulties with hand or finger movements);
- Significant scalp damage (i.e., preventing the use of a cap during the study);
- Regular use of medication.

### 2.2. Task Design

Prior to the commencement of the procedure, participants were provided with detailed information regarding the examination. Their task involved viewing a series of paired images. Following the introduction, the participant was invited into the laboratory, where they were seated in front of a desk equipped with an LCD monitor displaying the start screen. The participant was positioned approximately 50 cm from the screen. The fNIRS calibration procedure was then conducted. Optode positioning was also adjusted. Upon successful calibration, the researcher initiated the procedure in a shaded environment (due to the reduction of light interference, of which the subject had been informed) and remained in the laboratory for the duration of data collection.

The experiment began with a welcome screen, which included instructions outlining the purpose and procedure of this study, participants’ right to withdraw at any time, and assurances of data confidentiality. Following the welcome screen, participants viewed an example image pair. Next, a fixation point (a white ‘X’) appeared on the screen for 30 s to allow participants to stabilize their attention.

**Figure 2:**
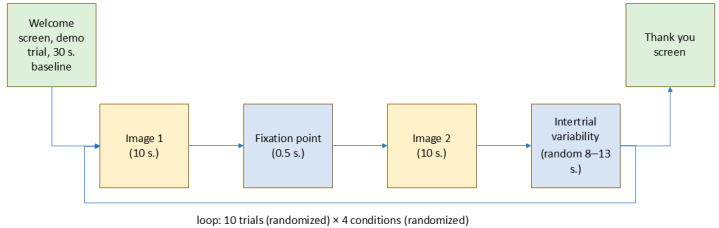
Schematic representation of the experimental procedure detailing trial sequences.

The core experimental phase then commenced, consisting of trials featuring image pairs. The sequence of images for each participant was randomized. Each trial lasted 26.5 s and was repeated 10 times. The timing and sequence of events followed the scheme outlined in Figure 2.

This study replicated the conditions of the original Jepma et al. [27] experiment, with some key modifications due to the use of fNIRS examination:

1. Increased stimulus presentation time. The reason for this modification was to capture a more complete hemodynamic response. While the original study employed 5 s stimulation blocks, the current protocol was adapted accordingly. Although there is evidence suggesting that a stimulation period of 15 s may be optimal for fNIRS measurements in general [53], we opted for a duration of 10 s. This decision was informed by the extended nature of the stimulation in our paradigm, as well as previous findings indicating that a 10 s stimulation window may be more appropriate for capturing responses in sensorimotor regions near the 2nd ROI [54,55].
2. Extended inter-trial variability. This adjustment was motivated by (1) the intention to ensure the complete resolution of the hemodynamic response between image pairs (trials) and (2) the lack of precise knowledge regarding the shape and duration of the HRF specific to this task. The selected inter-trial interval was therefore designed to hypothetically coincide with the post-stimulus undershoot phase of the hemodynamic response.
3. Reduction of the number of images to 80 distinct images and their blurred counterparts. We reduced the number of images due to the extended duration of stimulus presentation, with consideration for participant well-being during the session. On average, the stimulation phase lasted over 20 min.
4. No image repetition across or within conditions. As the number of images decreased, the probability of randomly selecting the same item from a closed set increased. To mitigate this, we opted to retain only unique, non-repeating images for each participant.

This study was conducted in accordance with the Declaration of Helsinki and approved by the University Research Ethics Committee of Maria Curie-Skłodowska University in Lublin (Decision No. 18/2023).

### 2.3. Probe Design of the fNIRS Cap

**Figure 3:**
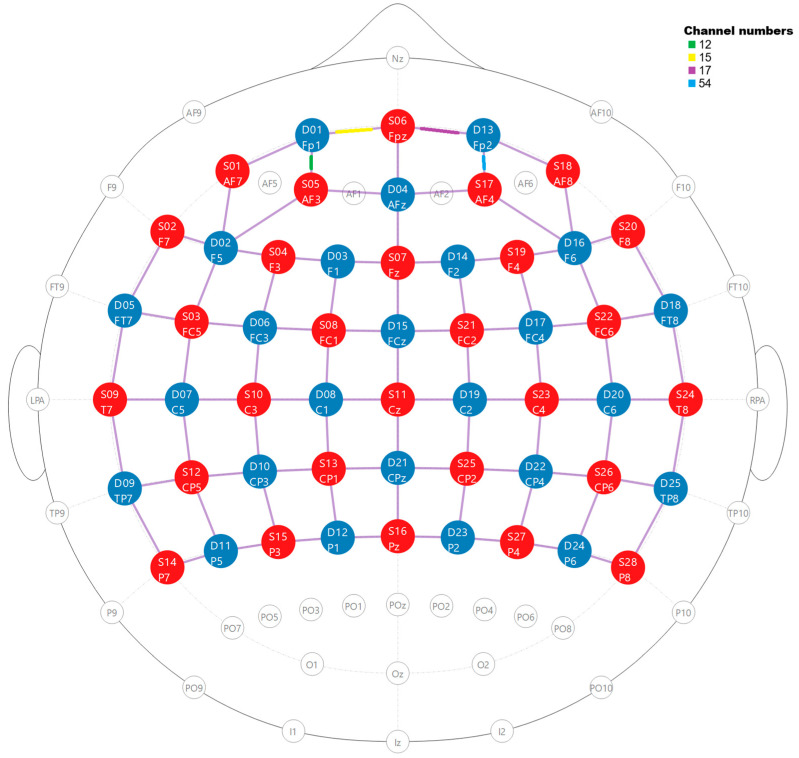
Location of sources (red) and detectors (blue) on the 2D surface. Numbers indicate the optode localization. Visualization obtained from the NIRSite application (abbreviations: Nz—nasion, Iz—inion, RPA—right pre-auricular, LPA—left pre-auricular).

The probe configuration consisted of 30 emitters and 22 detectors in a standard 10–20 EEG arrangement, covering the frontal, parietal, and temporal cortices. A total of 90 channels were obtained. The exact optode placements are provided in Appendix A. The distance between emitters and detectors was approximately 30 mm across all channels. The optode’s positioning was determined using predefined coordinates from the fOLD toolbox (version 2.2.1) [56] (see Figure 3 and Appendix A, Table A1). This tool uses precomputed simulations of photon migration to estimate the sensitivity of source–detector channels to specific cortical regions. It enables the anatomical evaluation of optode layouts by calculating how well each channel covers predefined brain areas. The tool supports multiple brain parcellation schemes; in our study, we used the Brodmann atlas [57] for anatomical referencing. For each source–detector pair, fOLD estimates photon penetration profiles across tissue layers, providing a specificity index (indicated in Table 1 for this study) that quantifies the channel’s sensitivity to individual cortical structures.

### 2.4. Signal Processing

The signal processing pipeline consisted of several steps. The analysis was conducted using Homer3 standalone software (v1.87.0) [58]. First, the raw fNIRS signal was converted into optical density using Beer–Lambert’s law. A bandpass filter was then applied (HPF: 0.010 Hz; LPF: 0.200 Hz) to remove low-frequency drifts and high-frequency noise. Motion artifacts were identified using changes in optical density data (tMotion = 0.5; tMask = 1; STDEVthresh = 50; AMPthresh = 5.0) and corrected with a spline interpolation method (*p* = 0.99). The optical density data were then converted into concentration changes (hmrR_OD2Conc function). Finally, the hemodynamic response function (HRF) was estimated using the general linear model (GLM) with the following parameters: trange: −2 to 10; glmSolveMethod: 1; idxBasis: 2; paramsBasis: 1.0, 1.0; rhoSD_ssThresh: 15.0; flagNuisanceRMethod: 0; driftOrder: 3; and c_vector: 0.

The signal quality was assessed using the QT-NIRS toolbox [59], applying SCI (0.8) and PSP (0.1) thresholds and with a minimum signal quality of 75%. Analyses were conducted in the MATLAB (R2023b) environment. Table 1 presents the percentage contribution of the acquired signal within channels in Brodmann areas.

## 3. Results

As part of the QT-NIRS analysis [59], one participant was excluded from further analysis due to low signal quality across the majority of channels. Additionally, channels located in the right and left parietal lobes were eliminated as they contained a high level of noise across the participant group.

**Figure 4:**
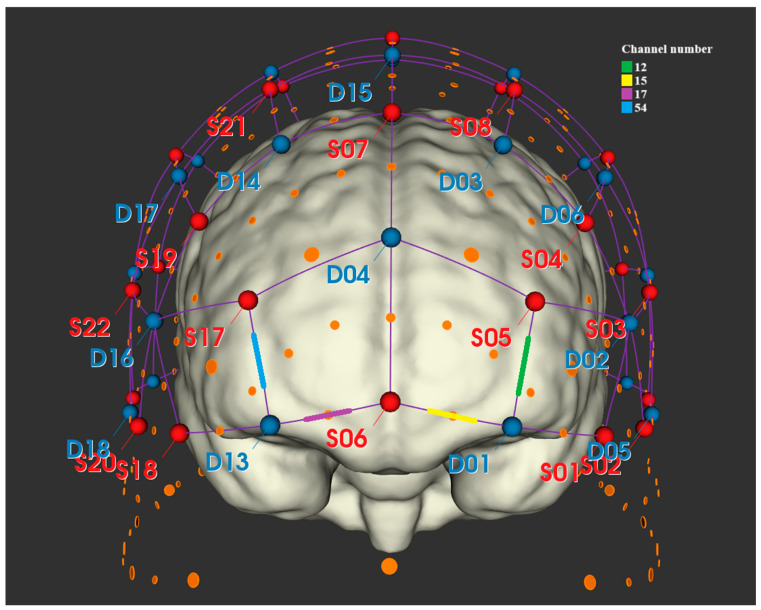
Location of channels on the 3D surface, including the brain model. The red marks (S08, among others) refer to the location of the sources, while the blue marks refer to the location of the detectors (D16, among others). The connections between them form channels. Visualization obtained from the NIRSite application.

Following these exclusions, 14 participants were retained for further analysis. The analyses focused on structures surrounding Brodmann Area 11 (BA11), which represents the most specific and fundamental region of the orbitofrontal cortex (OFC) according to the Brodmann classification. Activity in this brain region was recorded via channels 12, 15, 17, and 54 (see Table 1 and Figure 4). Additionally, a signal was recorded from other prefrontal areas, specifically the frontopolar prefrontal cortex (BA10), which encompasses the frontopolar prefrontal cortex, rostrolateral prefrontal cortex, and anterior prefrontal cortex.

To test this hypothesis, a paired-sample *t*-test was conducted using IBM SPSS Statistics 29. The analyzed dataset consisted of β values for Oxy-Hb, obtained as the output of the GLM signal-processing pipeline.

The general linear model (GLM) in fNIRS is used to model the hemodynamic response. It is based on the following equation:

*Y* = *Xβ* + *ε*

where *Y* represents the measured signal, *X* is the design matrix, *β* denotes the coefficients estimated by the model, and *ε* is the residual error. The modeling process begins by creating boxcar functions that describe the onset and duration of each stimulus. These functions are then convolved with a selected hemodynamic response function (HRF), reflecting the expected shape of the hemodynamic response over time. This convolution allows the model to account for the temporal dynamics of neurovascular coupling. The β values are estimated through model fitting and subsequently compared across conditions to identify significant effects [60,61].

In our study, the dependent variable was the change in oxyhemoglobin concentration. We accounted for signal drift. Experimental conditions were defined by event markers recorded during the task. To model the hemodynamic response, we used eight regressors corresponding to the onsets of stimuli (two images × four types of stimulation), as well as one regressor representing inter-trial interval periods.

Comparisons were performed between the first two experimental conditions following the unveiling of the second image:

1. Blurred image transitioning to a matching clear image;
2. Blurred image transitioning to a non-matching clear image.

The results of the paired-sample *t*-tests for each channel are presented in Table 2.

The results indicate a statistically significant difference in the mean β beta values of Oxy-Hb in channel 12. This suggests a differential hemodynamic response in the left dorsal prefrontal cortex (~20% of the signal variance originating from the OFC) between conditions involving satisfactory (matching) vs. unsatisfactory (non-matching) solutions of perceptual uncertainty.

The findings indicate a significant difference in the mean beta values of Oxy-Hb in channel 12. This observation suggests that OFC activity was higher in the condition where uncertainty was decreased (reduced curiosity).

## 4. Discussion

In the present study, we investigated differences in cortical activation during the viewing of pairs of images designed to induce a state of perceptual uncertainty. This study was a partial replication of the methodology applied by Jepma and colleagues [27], although it was adapted to use fNIRS as the measurement technique, along with an alternative analysis model. We hypothesized that differences in the hemodynamic response would be observed when comparing two blurred vs. non-blurred images (matching vs. not-matching conditions). The findings partially supported our hypothesis regarding differences in the activation of OFC and in adjacent structures encompassing BA10/11, proving the replicability of the fMRI procedure in the fNIRS environment.

The obtained results align with the existing literature, particularly in relation to the described neural pathways of curiosity, indicating the important role of OFC in curiosity [43]. The OFC plays a role in multiple processes, which may explain its involvement in the present study. The processes associated with activation of the OFC include emotion regulation [62,63] and reward processing [64]. Moreover, the OFC maintains strong connections with other key brain structures, including the striatal circuit (which overlaps with the previously mentioned curiosity pathway). This circuit is responsible for reward state representation, and its activation facilitates reward learning [65].

The OFC is a complex structure that appears to be strongly linked to curiosity pathways due to its involvement in learning, information processing, and executive functions [66]. Neuroimaging studies in primates suggest that the OFC primarily encodes variables relevant to learning, attention, and decision-making rather than integrating them. It is hypothesized that the OFC may regulate information-seeking behavior in response to internal states, such as uncertainty and curiosity [67]. The integration of reward value following state induction may occur in downstream structures, potentially including the vmPFC [68,69]. This observation is further supported by studies demonstrating OFC activation in the relief condition (following curiosity induction), similar to our study [49].

To explain how activation of the OFC is associated with curiosity reduction, we refer to data indicating that curiosity is a strongly rewarding emotion/a state that involves the reward system [64]. The OFC is a brain structure that is included in the reward system. Thus, it can be associated with maintaining motivation in the learning process. The orbitofrontal cortex (OFC) and prefrontal cortex (PFC) are engaged in the early stages of stimulus evaluation and decision-making processes. Activity in these regions—particularly when considering individual variability in the anterior cingulate cortex (ACC)—may influence motivational factors and the maintenance of readiness for learning and exploration. Some theoretical models of curiosity suggest that it may not solely reflect an intrinsic drive to resolve uncertainty, but rather functions to sustain learning processes, particularly when individuals perceive progress in their performance [70]. Within this framework, the role of the OFC in curiosity becomes especially relevant, as it may support the monitoring of reward-related signals and the adaptive regulation of learning behavior.

On the other hand, the OFC is not the dominant structure within channel 12 of the fNIRS (AF3-Fp1). In addition to the orbitofrontal cortex, most of the fNIRS signal in this channel originates from the prefrontal cortex, which includes areas such as the dorsolateral prefrontal cortex (DLPFC), dorsomedial prefrontal cortex (dmPFC), and ventromedial prefrontal cortex (vmPFC). Their activation in response to the induction of uncertainty (i.e., curiosity) suggests the involvement of affective memory regulation processes [45]. These processes may be particularly pronounced during the resolution of uncertainty compared to the non-matching condition (i.e., certainty vs. absence of curiosity).

Additionally, the OFC may not play a central role in decreasing uncertainty/reducing curiosity. In this aspect, OFC activity could be functionally associated with the hippocampus, which may contribute to this processing as it is involved in memory integration and decision-making processes [71]. Paradoxically, this could be relevant given the relatively long stimulus presentation time in our experimental study. The decrease in stimulus attractiveness over time might have led to changes in the intensity of activation in the measured brain regions, ultimately affecting the structure of the acquired data.

Our study also revealed a diminished manipulation effect, which could be attributed to two factors: (1) the lack of participant interaction with the environment and (2) the relatively long experiment duration. A similar mechanism is described in the work of Rankin et al. [72], which emphasizes the difficulty of maintaining sustained curiosity induction over extended periods. Curiosity is inherently challenging to study as habituation occurs—participants become accustomed to a new, initially attractive stimulus, rendering it no longer novel or engaging. Habituation may have led to the averaged deactivation of brain structures. If this explanation holds, future studies should focus on controlling signal drift or modifying the procedure to reduce the potential impact of habituation.

Alternatively, the reduced OFC activation observed in our study may suggest that other structures compensated for its role. Ferrari et al. [73] demonstrated that dorsolateral prefrontal cortex (DLPFC) stimulation (a region contributing to channel 12 variance in our study) via tDCS increased the perceived attractiveness of facial aesthetics when participants evaluated other faces. There is also known evidence that the amygdala plays a role in this process [74]. However, due to the limitations of fNIRS (e.g., relatively poor spatial resolution and the technical inability to capture the signals from subcortical structures), future studies should consider using alternative neuroimaging techniques to verify hypotheses related to curiosity measurement.

Referring to the factors that potentially modify the data obtained in the curiosity experiment, we will consider the importance of gender, age, and education.

The data expose us to the potential influence of individual variability, including the contribution of demographic factors. In our study, we observed a marked overrepresentation of female participants. A literature review conducted by Wagstaff et al. [75] reported mixed or nonsignificant gender differences in curiosity. Similarly, other studies suggest that curiosity—albeit variably defined—tends to be a relatively gender-neutral construct [60,76]. For this reason, we did not opt to limit our already small sample to a monogender group.

The relationship between curiosity and age presents a different pattern. Evidence indicates that curiosity tends to decrease with age [77]. This may be explained by the age-related decline in the cognitive resources necessary for engaging with curiosity-driven processing [77,78]. However, Heintz and Ruch [79], conceptualizing curiosity as one of the twenty-four personality strengths, found the opposite trend—curiosity increased with age. In our study, the sample (aged 21–30) is relatively homogenous and falls within the developmental stage of early adulthood, as framed by developmental theories and lifespan research traditions (e.g., Robinson and Smith [80]; Levinson [81]). Thus, we did not include age as a covariate in our analyses.

The relationship between curiosity and education level warrants more thorough investigations. While curiosity has been found to correlate with educational performance, findings on its link to educational attainment remain mixed. For example, Mussel [82] reported that curiosity predicted tertiary but not secondary educational attainment. Moreover, Fry, Elkins, and Farrel [83] found positive associations between curiosity and science and reading abilities, but a negative association with mathematics ability. Higher educational attainment has also been shown to predict enhanced cognitive performance and learning efficacy [84]. These findings suggest a potentially complex relationship between curiosity and educational background, which merits further exploration. Finally, research by Sternszus, Saroyan, and Steinert [85], conducted among medical students in their first to fourth years of study, indicates that curiosity remains relatively stable over time. At the same time, the authors emphasize the conceptual distinction between curiosity as a trait and as a state.

### Limitations

Our study is not free of limitations. It is important to note that most channels classified as analyzable in our study did not show significant differences in mean *β* values between conditions. One possible explanation is the relatively small sample size. Statistical analyses based on mean values are sensitive to individual variability, which is a common challenge in neuroimaging studies where collecting large samples can be difficult.

Additionally, the exclusion of the inferior parietal lobule as a region of interest (ROI) due to unsatisfactory signal quality may have impacted the results, as this region was originally a primary target of examination. Poor measurement quality in some regions may also have stemmed from suboptimal cap fitting for certain participants or insufficient sampling frequency, potentially causing aliasing artifacts, as discussed by Tong and associates [86].

The correction for multiple comparisons is a standard procedure in confirmatory research approaches. However, given the exploratory and pilot nature of our study, we emphasize that the findings should not be generalized to the broader population. In particular, we note that the observed *p*-value of 0.04 should be interpreted with caution, as it lies near the conventional threshold for significance. Due to the relatively small sample size, we applied a more liberal statistical threshold in this preliminary investigation. Nevertheless, for future studies, we recommend adopting a more conservative approach (e.g., *p* < 0.01) to ensure the greater robustness of the findings.

The main contribution of this article lies in the successful adaptation of an fMRI-based paradigm in an fNIRS setting. Additionally, our results highlight the potential of this approach to inform hypothesis-driven research in studies with larger sample sizes.

## 5. Conclusions

Given the pilot study nature of this research, the obtained results should be interpreted with caution. The findings did not fully confirm all hypotheses, yet they provide valuable insights into the direction of future research and experimental design for curiosity studies.

We confirmed higher OFC activity in conditions associated with decreasing perceptual uncertainty (reduced curiosity), consistent with the existing body of literature. Our additional conclusions concern the design of curiosity-inducing experiments. We believe that implementing modifications, such as reducing the number of trials and introducing interactive elements during the experiment, may positively impact participant well-being and, in turn, reduce habituation effects in laboratory-based curiosity research.

## Figures and Tables

**Table 1:** Localization of individual channels included in the analyzed regions of interest (ROI).

Channel	Source	Detector	BA11 Specificity	BA10 Specificity	BA46 Specificity	Hemisphere
12	AF3	Fp1	20.12%	69.63%	8.79%	Left
54	AF4	Fp2	21.57%	68.78%	6.61%	Right
15	Fpz	Fp1	44.90%	54.50%	n/a	Left
17	Fpz	Fp2	44.85%	54.46%	n/a	Right

Note. Abbreviations: n/a—not applicable. All of the above channels mostly cover the BA10 area. Channel localization and specificity are created using the fOLD tool [56].

**Table 2:** Results of the paired-sample *t*-tests for the unveiling conditions.

Channel	Corr M *β*	Ncorr M *β*	SD	*T*	*df*	Two-Tailed *p*-Value
12	−0.000032959	−0.000118106	0.000122152	2.284	13	0.04
54	−0.000020185	−0.000174224	0.000188508	0.117	13	0.91
15	−0.000082886	−0.000017293	0.000129915	1.320	13	0.21
17	−0.000094318	−0.000026609	0.000141584	−1.050	13	0.31

Note. Abbreviations: M—mean *β* values, SD—standard deviation.

## Data Availability

The original contributions presented in this study are included in the article. Further inquiries can be directed to the corresponding author.

## Abbreviations

The following abbreviations are used in this manuscript:

fNIRS	Functional near-infrared spectroscopy
OFC	Orbitofrontal cortex
PFC	Prefrontal cortex
IFG	Inferior frontal gyrus
ACC	Anterior cingulate cortex
IPL	Inferior parietal lobule
LHb	Lateral habenula
DAM	Dopaminergic midbrain
NAcc	Nucleus accumbens
rlPFC	Rostrolateral prefrontal cortex
vmPFC	Ventromedial prefrontal cortex
OTC	Occipitotemporal cortex
LOC	Lateral occipital cortex
EEG	Electroencephalography
HPF	High pass filter
LPF	Low pass filter
HRF	Hemodynamic response function
GLM	General linear model
SCI	Scalp coupling index
PSP	Peak spectral power
ROI	Region of interest
BA	Brodmann area

## Appendix A

**Table A1:** Table of NIRS channel localizations.

Channel No.	Source	Detector	Position X	Position Y	Position Z
1	1	1	−42.889	78.096	−16.978
2	1	2	−59.589	60.168	−4.362
3	2	2	−68.363	46.595	−4.375
4	2	5	−76.056	28.17	−18.528
5	3	2	−72.412	34.597	14.859
6	3	5	−78.803	16.889	0.924
7	3	6	−71.835	22.115	37.64
8	3	7	−81.342	2.605	21.617
9	4	2	−58.802	54.143	23.513
10	4	3	−41.387	58.42	48.855
11	4	6	−58.817	39.436	45.984
12	5	1	−31.819	82.022	0.591
13	5	2	−52.105	65.735	13.046
14	5	4	−18.354	83.517	24.189
15	6	1	−14.887	88.69	−10.565
16	6	4	0.084	87.747	11.946
17	6	13	15.353	88.52	−10.555
18	7	3	−15.837	63.087	62.131
19	7	4	0.193	75.25	49.43
20	7	14	14.824	63.339	62.24
21	7	15	−0.417	47.185	78.028
22	8	3	−33.621	44.199	68.798
23	8	6	−51.155	26.914	66.065
24	8	8	−38.473	8.634	84.604
25	8	15	−19.002	29.518	84.724
26	9	5	−82.152	−0.704	−17.684
27	9	7	−85.287	−14.179	3.539
28	9	9	−87.447	−30.648	−14.486
29	10	6	−67.497	6.477	58.072
30	10	7	−80.047	−11.793	44.03
31	10	8	−57.076	−9.562	77.439
32	10	10	−72.341	−29.617	65.522
33	11	8	−20.29	−8.513	97.94
34	11	15	−0.033	11.7	95.025
35	11	19	19.132	−8.372	98.259
36	11	21	−1.124	−29.306	103.979
37	12	7	−85.028	−29.926	25.727
38	12	9	−83.896	−46.505	6.45
39	12	10	−78.594	−47.958	46.962
40	12	11	−77.374	−64.104	26.858
41	13	8	−39.895	−28.88	93.294
42	13	10	−56.069	−49.118	80.686
43	13	12	−37.079	−66.678	87.378
44	13	21	−20.119	−48.819	100.055
45	14	9	−79.372	−61.256	−10.554
46	14	11	−72.834	−77.39	8.481
47	15	10	−65.587	−64.985	61.605
48	15	11	−65.27	−80.772	41.942
49	15	12	−46.473	−83.647	67.196
50	16	12	−16.52	−83.46	82.647
51	16	21	−0.559	−67.109	96.113
52	16	23	15.737	−83.958	82.348
53	17	4	18.291	83.473	24.403
54	17	13	32.689	81.589	0.629
55	17	16	51.371	66.117	14.439
56	18	13	42.847	77.838	−15.335
57	18	16	59.018	60.776	−3.202
58	19	14	41.232	59.179	48.129
59	19	16	58.579	53.906	24.739
60	19	17	58.283	40.934	45.288
61	20	16	68.187	46.903	−4.129
62	20	18	75.594	29.539	−17.618
63	21	14	32.853	44.739	68.91
64	21	15	17.84	29.239	85.234
65	21	17	49.824	27.354	67.243
66	21	19	37.492	9.942	84.648
67	22	16	72.157	35.093	15.105
68	22	17	71.344	23.106	37.681
69	22	18	78.527	18.038	1.437
70	22	20	80.866	4.38	23.264
71	23	17	66.337	8.158	58.736
72	23	19	55.859	−8.634	78.224
73	23	20	79.353	−10.899	45.643
74	23	22	71.228	−27.778	67.002
75	24	18	81.902	0.854	−15.968
76	24	20	85.078	−12.698	4.825
77	24	25	86.471	−30.117	−13.132
78	25	19	39.401	−28.293	93.506
79	25	21	20.095	−48.636	100.077
80	25	22	54.798	−47.767	82.138
81	25	23	37.194	−66.437	87.445
82	26	20	84.951	−28.595	27.436
83	26	22	78.473	−47.501	47.691
84	26	24	78.121	−62.051	27.562
85	26	25	83.928	−46.19	8.435
86	27	22	65.533	−64.259	62.278
87	27	23	46.356	−82.981	68.005
88	27	24	66.047	−79.714	41.904
89	28	24	73.274	−76.332	9.546
90	28	25	79.743	−60.24	−9.105

**Table A2:** List of images used in the stimulus procedure. Obtained from the Rossion and Pourtois set [52].

Index	Condition	1-st Image	2-nd Image
1	Blurred—clear (corresponding)	Zebra (blurred)	Zebra
2		Fence (blurred)	Fence
3		Padlock (blurred)	Padlock
4		Anchor (blurred)	Anchor
5		Brush (blurred)	Brush
6		Desk (blurred)	Desk
7		Turtle (blurred)	Turtle
8		Bee (blurred)	Bee
9		Guitar (blurred)	Guitar
10		Heart (blurred)	Heart
1	Blurred—clear (unrelated)	Eye (blurred)	Candle
2		Pants (blurred)	Cake
3		Swing (blurred)	Rabbit
4		Lobster (blurred)	Celery
5		Saw (blurred)	French horn
6		Butterfly (blurred)	Ladder
7		Sweater (blurred)	Leopard
8		Elephant (blurred)	Book
9		Grapes (blurred)	Rhino
10		House (blurred)	Grasshopper
1	Clear—blurred	Winter glove	Winter glove (blurred)
2		Strawberry	Strawberry (blurred)
3		Caterpillar	Caterpillar (blurred)
4		Barn	Barn (blurred)
5		Lemon	Lemon (blurred)
6		Wooden wheel	Wooden wheel (blurred)
7		Umbrella	Umbrella (blurred)
8		Eagle	Eagle (blurred)
9		Rooster	Rooster (blurred)
10		Thread on bobbin	Thread on bobbin (blurred)
1	Clear—clear	Watch	Watch
2		Chair	Chair
3		Watering can	Watering can
4		Harp	Harp
5		Salt shaker	Salt shaker
6		Pen	Pen
7		Long-sleeve shirt	Long-sleeve shirt
8		Sofa/couch	Sofa/couch
9		Scissors	Scissors
10		Pineapple	Pineapple
